# Magnetic Solid Phase Extraction Based on Nanostructured Magnetic Porous Porphyrin Organic Polymer for Simultaneous Extraction and Preconcentration of Neonicotinoid Insecticides From Surface Water

**DOI:** 10.3389/fchem.2020.555847

**Published:** 2020-09-16

**Authors:** Shirley K. Selahle, Ngwako J. Waleng, Anele Mpupa, Philiswa N. Nomngongo

**Affiliations:** ^1^Department of Chemical Sciences, University of Johannesburg, Doornfontein Campus, Doornfontein, South Africa; ^2^Department of Science and Innovation/National Research Foundation South African Research Chairs Initiative Chair: Nanotechnology for Water, University of Johannesburg, Doornfontein, South Africa; ^3^Department of Science and Innovation/Mintek Nanotechnology Innovation Centre, University of Johannesburg, Doornfontein, South Africa

**Keywords:** neonicotinoid insecticides, porphyrin based organic polymer, high performance liquid chromatography, adsorption mechanism surface water, magnetic solid-phase extraction

## Abstract

In this study, a magnetic porphyrin-based porous organic polymer (MP-POP) nanocomposite was successfully synthesized according previous studies and applied as an adsorbent for simultaneous extraction and preconcentration of four neonicotinoid insecticides from surface river water. The MP-POP was characterized using X-ray diffraction (XRD), transmission electron microscopy (TEM), scanning electron microscopy/energy dispersive x-ray spectroscopy (SEM/EDS), N_2_-adsorption/desorption analysis, Fourier Transform infrared spectroscopy (FTIR). The neonicotinoid insecticides were quantified using high performance chromatography coupled with diode array detector (HPLC-DAD). The MP-POP shown to have a high surface area, highly porous structure and strong affinity toward the investigated analytes. The adsorption capacities were 99.0, 85.5, 90.0, and 79.4 mg g^−1^ for acetamiprid, clothiandin, thiacloprid and imidacloprid, respectively. The influential parameters affecting the magmatic μ-solid phase extraction (M-μ-SPE) procedure were investigated using fractional factorial design and surface response methodology (RSM). Under optimum conditions, the method exhibited relatively low limit of detection in the range of 1.3–3.2 ng L^−1^, limit of quantification in the range of 4.3–11 ng L^−1^ and wide linearity (up to 600 μg L^−1^). The intraday and interday precision, expressed as the relative standard deviation (RSD) were <5%. The percentage recoveries for the four target analytes ranged from 91 to 99.3% for the spiked river water samples. The method was applied for determination of neonicotinoids in river water samples and concentrations ranged from 0 to 190 ng L^−1^.

## Introduction

Neonicotinoid insecticides are applied worldwide to combat unwanted insects from attacking crops which led to their entry into the environment (Goulson, 2013; Klarich et al., 2017; Hladik et al., 2018). However, over- application of neonicotinoid insecticides in the environment can cause negative effects on human health and living organisms (Klarich et al., 2017; Struger et al., 2017). Several severe human diseases such as cancer, chronic pulmonary disease, birth defects and infertility are associated with the exposure to neonicotinoid insecticides (Giroud et al., 2013; Sánchez-Bayo and Hyne, 2014; Vichapong et al., 2015). The permissible maximum residue limits for neonicotinoid insecticides have been controlled by the Codex Alimentarius Commision and European Union and World Health Organization to protect human health (Vichapong et al., 2016). However, these regulated limits are only applicable to crops and soil, not to portable water, river water, water reservoirs and surface or ground water (Vichapong et al., 2015, 2016). Therefore, to ensure safe water to humans and living organisms, an effective technique for the detection of the neonicotinoid insecticides in surface water, portable water, river water and water reservoirs is of remarkable significance.

Different types of analytical techniques including gas chromatography (Ai et al., 2010; Kiljanek et al., 2016; Balsebre et al., 2018), high performance liquid chromatography (HPLC) (Wu et al., 2011; Giroud et al., 2013; Vichapong et al., 2015; Cao et al., 2018; Kachangoon et al., 2020) and liquid chromatography tandem mass spectrometry (LC-MS/MS) (Bolzan et al., 2015; Kiljanek et al., 2016; Zhou et al., 2018; Hou et al., 2019) have been used for the detection and analysis of neonicotinoid insecticides in various samples. However, due to the intricacy of different sample matrices and the trace amounts of the neonicotinoid insecticides, sample clean-up techniques are required prior to instrumental analysis. The benefits of sample preparation do not only involve sample clean-up, but also preconcentration of the target analytes (Farajzadeh et al., 2016; Vichapong et al., 2016; Moyakao et al., 2018; Kachangoon et al., 2020). Currently, several sample extraction techniques, such as cloud point extraction (CPE) (Kachangoon et al., 2020), liquid-phase microextraction (LLME) (Zhang et al., 2012; Jovanov et al., 2013; Bolzan et al., 2015; Vichapong et al., 2015; Hou et al., 2019; Kachangoon et al., 2020) solid-phase extraction (SPE) (Xie et al., 2011; Shi et al., 2017; Zhang et al., 2017; Cao et al., 2018; Moyakao et al., 2018; Xiong et al., 2018; Hou et al., 2019) and solid-phase microextraction (SPME) (Ding et al., 2019; Queiroz et al., 2019; Xue et al., 2019), have been reported for the extraction of the neonicotinoid insecticides in various samples. Among the SPE based methods magnetic solid-phase extraction (MSPE) has gained a lot of attention due to its attractive properties such as simplicity, rapidity, robustness, high enrichment factors, and environmentally friendliness (Deng et al., 2009, 2019; Wang et al., 2018b; Queiroz et al., 2019). In MSPE, the magnetic sorbent play a major role on the analytical performance of the method (He et al., 2014; Fumes et al., 2015; Jiang et al., 2019; Li and Shi, 2019). Therefore, it is important to design, synthesize, and explore efficient magnetic adsorbents with high affinity toward target analytes.

Recently, literature has suggested that porous adsorbents such as porous organic polymers (POPs) have received significant attention in different scientific field. They have been applied as novel materials in luminescent sensing (Li et al., 2019; Pan et al., 2019) catalysis (Kaur et al., 2011; Zhang and Riduan, 2012; Das et al., 2019; Li et al., 2020), gas storage (Wood et al., 2008; Das et al., 2019), electrochemical sensing (Vilian et al., 2018), drug delivery, and adsorption and SPE (Huang et al., 2017; Wang et al., 2017, 2018a,b; Xiong et al., 2019; Li et al., 2020). These materials are prepared by combining various monomer units using different types of chemical reactions (Li et al., 2020). Depending on the type of chemical reaction, a wide range of structural frameworks are achieved (Wood et al., 2008; Wang et al., 2017, 2018a,b; Li et al., 2020). Structurally, POPs poses strong covalent bonds that leads to attractive features such as high mechanical, chemical and thermal stabilities (Wang et al., 2017, 2018a,b). Among different types of porous materials, the application of porphyrin based POPs (PPOPs) as adsorbents is increasing due to their large macrocyclic cavity, surface areas, remarkable stability and excellent affinity toward organic pollutants (Wang et al., 2017, 2018a,b). However, little has been reported about their application as adsorbent in sample preparation methodologies. Owing to the attractive features of P-POPs materials, they serves as suitable sorbents for extraction and preconcentration of low concentration neonicotinoid insecticides in various matrices.

Previous studied on sample preparation methods have applied once factors at time approach for the optimization of factors affecting preconcentration of target analytes (Asadollahzadeh et al., 2014). However, this approach is time-consuming, laborious, and sometimes unable to reach the accurate optimum because the interactions among the investigated variables are ignored (Asadollahzadeh et al., 2014). To overcome these challenges, optimization of factors affecting different variables has been achieved using design of experiments (DOE). This approach allows minimal number of with of experimental runs, thus leading to cost-effective method and acceptable results (Asfaram et al., 2017). In addition, DOE permits the investigation of interaction among the selected variables (Asfaram et al., 2017; Bagewadi et al., 2018). The use of DOE models such as Plackett–Burman design (PBD), full factorial design, fractional factorial design and response surface methodology (RSM) [such as Box–Behnken design, Doehlert design, central composite design (CCD)] has been reported in the literature (Zolgharnein et al., 2013; Asadollahzadeh et al., 2014; Benredouane et al., 2016; Asfaram et al., 2017; Bagewadi et al., 2018). Among the above-mentioned RSM models, CCD, is one of the most effective experimental designs. This is because CCD allows each independent variable to be investigated at five levels (that is, two-level factorial (±1), axis points (±α) and central points (0) (Mousavi et al., 2018). In addition, in cases where many variables need to be optimized, screening designs such as full or fractional factorial designs are mostly applied (Asadollahzadeh et al., 2014; Benredouane et al., 2016).

The aim of this study was to prepare magnetic porphyrin-based porous organic polymer (MP-POP) nanocomposite as a magnetic adsorbent in ultrasound assisted dispersive magnetic solid phase extraction (UA-DMSPE) and preconcentration of neonicotinoid insecticides from river water samples. The neonicotinoid insecticides in samples were quantified using HPLC-DAD. Previous studies reported the application MP-POP as an adsorbent for preconcentration of nitrogen bearing analytes such as benzoylurea insecticides and phenylurea herbicides and the result revealed that MP-POP has high affinity toward the target analytes (Wang et al., 2017, 2018a,b). Therefore, the adsorbent was chosen because of its high adsorption capacity, high surface area and strong affinity to aromatic compounds containing nitrogen atoms. The MP-POP nanocomposite good magnetic properties and could be easily separated from aqueous solution by an external magnet. The factors affecting the extraction and preconcentration procedure were optimized using fractional factorial design (FFD) and central composite design (CCD). These multivariate approach chosen because they can reduce the number of experiments required and gives more quantitative information about the significance of independent variable and their interactions (Bezerra et al., 2019; Tan and Lee, 2019). FFD was used screening was carried out to obtain the critical parameters for the extraction and preconcentration of neonicotinoid insecticides The UA-DMSPE/HPLC-DAD procedure was successfully used for simultaneous extraction, preconcentration, separation, and quantification of neonicotinoid insecticides in river water samples.

## Experimental

### Reagents and Standards

Terephthalaldehyde (98%), Pyrrole (99%), Ferrous chloride tetrahydrate (FeCl_2_·4H_2_O), Ferric chloride hexahydrate (FeCl_3_·6H_2_O), and aqueous ammonia (w, 30%), tetrahydrofuran (THF), dichloromethane, glacial acetic acid and HPLC grade methanol (99, 9%) and acetonitrile (99, 9 %) were all purchased from Sigma-Aldrich (St Louis, MO, USA). The analytes were reagent grade insecticides (clothiandin, imidacloprid, acetamiprid, and thiacloprid) were procured from Sigma-Aldrich (South Africa) Ltd. A 10 mg L^−1^ stock solution containing the analytes was prepared by weighing and dissolving the insecticides in acetonitrile. The stock solution was transferred in amber storage bottles and stored at 4°C before use. Working synthetic samples were prepared daily by diluting appropriate volumes of the stock solution with ultra-pure water (Direct-Q 3 UV-R purifier).

### Sampling and Sample Collection

River water samples were collected in different points at Apies River (Pretoria, Gauteng, South Africa). Schott bottles with caps (500 mL) were cleaned and used to collect water samples. When not in use, the samples were stored at 4°C.

### Instrumentations

The functional groups and structural changes of the nanocomposite were investigated using on a Perkin-Elmer Spectrum 100 spectrometer (Perkin-Elmer, USA). The samples were mixed with the potassium bromide (KBr) for form pellet and the spectra were recorded in the 400–4,000 cm^−1^ region. The morphological structure and elemental composition of the nanocomposite was were assessed using scanning electron microscopy equipped with energy dispersive X-ray spectroscopy (EDS) at a voltage of 20 kV (SEM, TESCAN VEGA 3 XMU, LMH instrument (Czech Republic). The internal structure of the composite was studied by dispersing the adsorbent in methanol, transferring a drop of the mixture onto a copper grid and analysis using transmission electron microscopy at a voltage of 120 kV (TEM JOEL JEM-2,100, Japan). The crystal structure and pore size distribution were studied using X-Ray diffraction (XRD) and BET N_2_ adsorption, respectively, Analyzer (ASAP2020 V3. 00H, Micromeritics Instrument Corporation, Norcross, USA). The Barrerr-Joyner-Halenda method was used to calculate the adsorbent pore volumes. An OHAUS starter 2,100 pH meter (Pine Brook, NJ, USA) for pH adjustments of reagents and pH of samples. Chromatographic analysis was carried out at wavelengths 250 nm and 260 nm using an Agilent HPLC 1,200 infinity series, with a diode array detector (Agilent technologies, Waldbronn Germany) and a Agilent Zorbax Eclipse Plus C18 column (3.5 μm × 150 mm × 4.6 mm) (Agilent Newport, CA, USA) baked at oven temperature of 25°C. All quantifications were done using an isocratic elution programme with mobile phase system containing water (mobile phase A, 70%) and acetonitrile (mobile phase B, 30 %) at a flow rate of 0.1.00 mL min^−1^.

### The Preparation of MP-POP Nanocomposite

Magnetic porphyrin based porous organic polymer was synthesized following methods described by Wang et al. (2017, 2018a,b). The preparation of MP-POP was a two-step process where the porphyrin based porous organic polymer was synthesized in step 1, followed by co-precipitation in step two to make the MP-POP.

#### Preparation of the P-POP

The synthesis of P-POP was carried out as follows: ~0.10 g of fresh pyrrole and 0.20 g terephthaldehyde were placed into a dry round bottom flask containing glacial CH_3_COOH (50 mL) and iron(III) chloride (0.47 g). The mixture was then agitated using a magnetic stirrer bar under a gentle stream of nitrogen for 3 h. The resultant solution was gentle transferred into a Teflon lined autoclave. The mixture in the autoclave was hydrothermal treated by placing the autoclave in an oven at 180°C for 24 h. The reaction vessel (autoclave) was cooled at room temperature and the resultant dark brown precipitate was separated from aqueous solution by centrifugation. The solid product was with water, methanol and tetrahydrofuran (THF), respectively, and it was dried under vacuum at 70°C.

#### The Preparation of the MP-POP

1.17 g FeCl_3_·6H_2_O and 0.43 g FeCl_2_·4H_2_O were placed in a round bottom flask having 500 mg of P-POP and 250 mL of water and mechanically stirred at room temperature under N_2_ atmosphere. The solution was then heated to 50°C followed by the dropwise addition of 14% ammonia solution under a pH of 11-12 was achieved. The reaction was continued for an hour to allow for the complete growth of nanoparticles. Magnetic separation was then used to collect the final product which was washed with deionised water several times until pH 7 and then with ethanol before finally being dried at 60°C.

### Ultrasound Assisted Dispersive Magnetic Solid Phase Extraction Procedure

A suitable amount of MP-POP nanocomposite was placed into 10 mL glass sample bottles having 5 mL of synthetic sample (containing a mixture of clothiandin, thiacloprid, acetamiprid and imidacloprid all at 100 μg L^−1^). The mixtures were then sonicated in an ultrasound water bath for 10–15 min. Magnetic separation done to separate the supernatant from the adsorbent. The supernatant was then decanted, and the analytes were eluted by sonicating the adsorbent with 500–100 μL of 100% acetonitrile for 3 min. The analysis of the analytes concentrations was done by the employment of the HPLC-DAD.

### Optimisation of UA- DMSPE Procedure

The most significant independent variables affecting the UA- DMSPE method were optimized using design of experiments (DOE). The firstly, screening of the parameters was done using the multivariate optimisation approach, specifically fractional factorial design (2^4−1^) and central composite design was carried out to determine the optimum conditions, and the parameters involved in the design are shown in Supplementary Table 1. Under optimum conditions, the method was applied investigate the effect of sample volume (5–50 mL) and initial concentration (50–2,000 μg L^−1^).

### Validation of the Method and Quality Assurance/Quality Control

The established method performance was validated by evaluating the accuracy (recovery), precision (intraday (repeatability) and interday (reproducibility), the linear dynamic range, preconcentration factor, enrichment factor and limit of detection (LOD) and limit of quantification (LOQ). The accuracy was evaluated for the target neonicotinoids in river water samples spiked at three concentration levels, 5, 100, and 500 μg L^−1^. The linearity was investigated by construction a seven-points calibration curve (the standards were prepared by spiking ultrapure water with a mixture of target analytes at 0 to 1,200 μg L^−1^). The LOD and LOQ were calculated as: LOD = ${\;}^{3Sd}{/}_{b}$ and LOQ = ${\;}^{10Sd}{/}_{b}$, where *Sd* is the standard deviation of 10 replicate determinations of the lowest concentration of calibration curves (0.5 μg L^−1^) and *b* is the slope of each calibration curve. In addition, the LOD and LOQs were determined using spiked blank river water samples with decreasing concentrations (0.01, 0.05, and 0.10 μg L^−1^) of each target analyte. For this purpose river water sample free from the target analytes was used. Linearity was performed using matrix-matched calibration curves. The blank river water sample was spiked with 0–700 μg L^−1^ solutions containing all target analytes. The sample were then processed with the developed method and seven point calibration was constructed. The repeatability and reproducibility of the developed method were calculated from several measurements of 5.0, 100, and 500 μg L^−1^ in river water samples. This was carried out to assess the matrix effect on the extraction efficiency of the developed method. The preconcentration factors were explained as the ratios between sample volume (Vs) and eluent volume (Ve) (Equation 1):

(1) $$\begin{array}{l} PF=V_{s}/V_{e} \end{array}$$

The enrichment factors (EF) was estimated as the fraction between the slopes before and after preconcentration (Equation 2):

(2) $$\begin{array}{l} EF=S_{ap}/S_{bp} \end{array}$$

Where Sap = slope after preconcentration; bp = slope before preconcentration.

In order to comply with the quality assurance/quality control (QA/QC) guidelines, blanks were injected to the HPLC-DAD system before the injection of any sample containing analytes. The chromatograms of the blank samples revealed that the samples (blanks) were free of target analytes. This indicated that the blank correction of the results obtained from samples containing the analytes was not required. Standard solutions of neonicotinoid insecticides at concentration of 50 and 500 μg L^−1^ were used as used as QA/QC samples. In the course of sample analysis, procedure blank samples (treated the same way as samples) and QA/QC standard solutions were analyzed after every ten samples.

### Adsorption Capacity and Regeneration Studies

The adsorption equilibrium experiments were conducted as follows: briefly 15 mg of MP-POP was weighed and transferred into 10 sealable glass containers. Then 30 mL of stock solution with varying concentrations (2–50 mg L^−1^) were then added into the glass bottle containing the adsorbent. Agitation of the solution by a sonicator was performed for 5 min. The absorbent and the supernatant were separated with the aid of an external magnet. Analysis of the supernatant was then carried out using HPLC-DAD. The adsorbent reusability was investigated by a series of extraction, elution, washing and drying. 15 mg of the adsorbent was added into a glass bottle container, then 30 mL of the sample was added into the glass container with adsorbent. The solution was sonicated for 15 min, then the supernatant and the absorbent were separated with the magnet. Elution was then carried out using acetonitrile, lastly the elute was then analyzed using HPLC-DAD. After each analysis the adsorbent was dried and reused.

## Results and Discussion

### Characterization of the Nanocomposite

**Figure 1:**
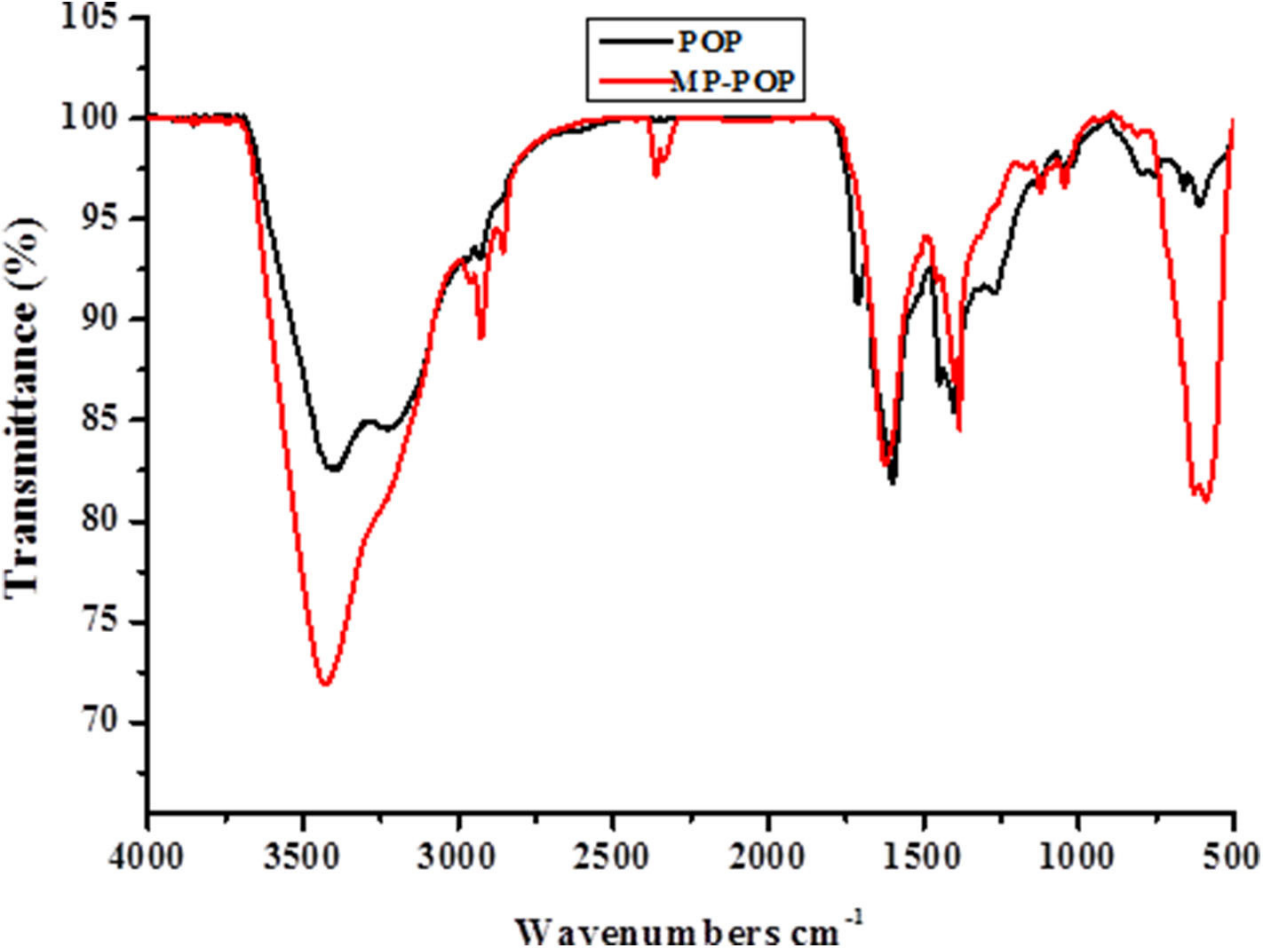
FTIR spectra of POP and MP-POP.

The functional groups of the synthesized MP-POP were confirmed using FTIR spectroscopy (Figure 1). The broad peak that appeared in both spectra at around 3,500 cm^−1^ indicates the N-H peak, and then followed by a smaller and sharp peak at around 2,986 cm^−1^ which is for phenyl (Wang et al., 2017, 2018a,b)A peak for alkene carbons (C=C) in the pyrrole ring was observed at 1,604 cm^−1^ and Fe-N peak at 1,047 ${\text{cm}}_{.}^{-1}$ The Fe-N peak shows that the magnetic iron was successfully incorporated on the POP without any alterations (Wang et al., 2017, 2018a,b). Lastly, a strong signal seen at around 570 cm^−1^ for Fe-O indicates the adherence of Fe_3_O_4_ on the surface of the POP (Ai et al., 2010; Wang et al., 2017). These verdicts were in line with those shown in the literature (Wang et al., 2017, 2018a,b).

**Figure 2:**
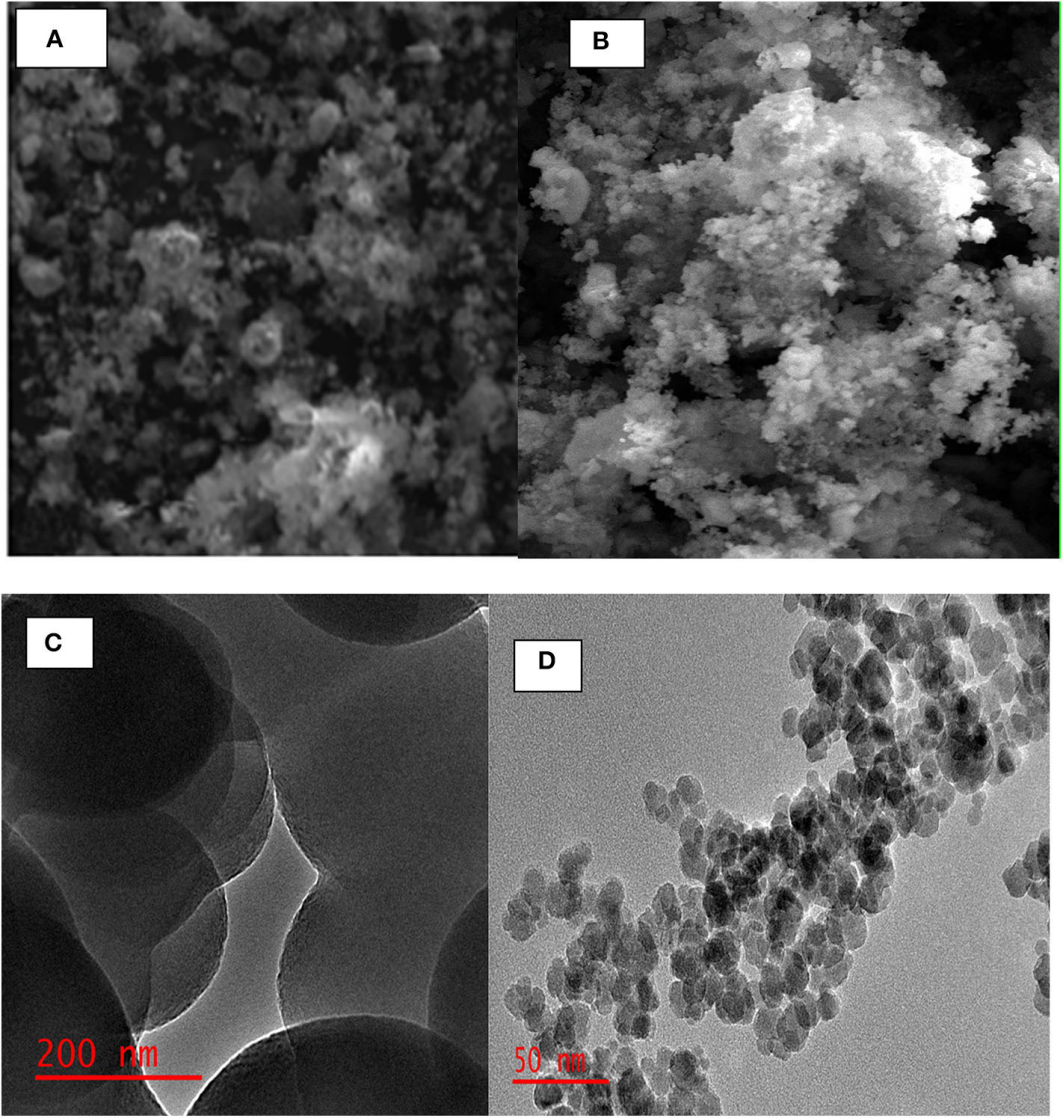
SEM images of **(A)** P-POP, **(B)** MP-POP and TEM images of **(C)** P-POP **(D)** of the M-PPOP.

Scanning electron microscopy and TEM were employed to investigate the porosity and morphology of POP and MP-POP nanomaterials. The SEM images (Figures 2A,B) displays that highly porous materials were successfully synthesized. The TEM image (Figure 2C) revealed that P-POP composed of was spherical shapes. Furthermore, Figure 2D revealed that that the Fe_3_O_4_ nanoparticles were successfully incorporated on the surface of P-POP (Wang et al., 2017, 2018a,b).

**Figure 3:**
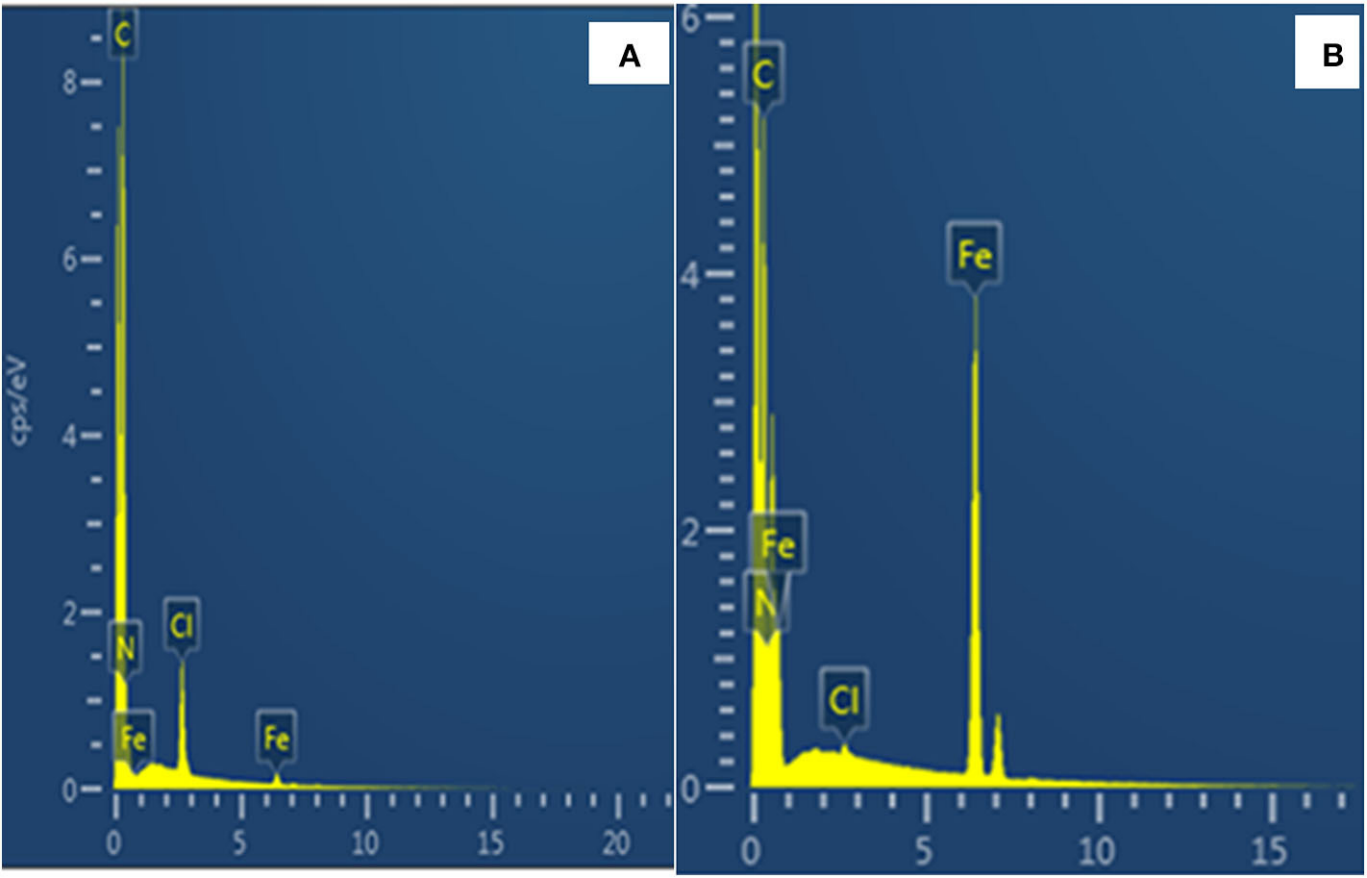
EDS spectra of **(A)** P-POP and **(B)** MP-POP.

The energy dispersive x-ray spectroscopy (EDS) spectra for POP and MP-POP are displayed in Figure 3. As seen in Figure 3A, as expected the spectrum for POP revealed presence of the N and C in the structure of POP. The presence of Cl and Fe was from the ferric chloride which was used in the synthesis. Figure 3B revealed all major elements present in MP-POP nanocomposite. The intense peak of Fe indicates that the Fe_3_O_4_ was successfully incorporated.

**Figure 4:**
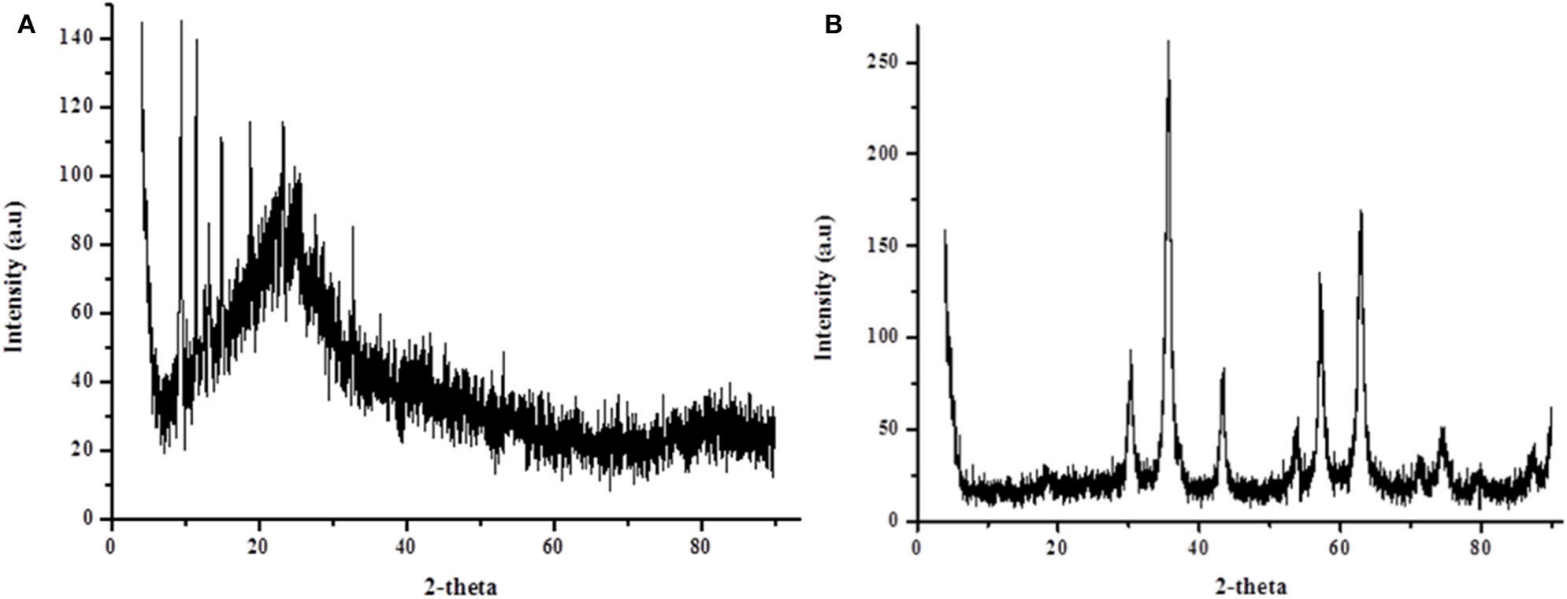
XRD patterns for **(A)** P-POP and **(B)** MP-POP.

The XRD spectroscopy was used to study the crystalline structure of P-POP and MP-POP. Figure 4A demonstrated that P-POP had weak diffraction peak around 2θ = 24° suggesting that the material was naturally amorphous (Wang et al., 2017, 2018a,b). The XRD patterns for MP-POP (Figure 4B) showed crystalline structure confirming the incorporation of magnetic particles on the surface of P-POP. The prominent diffraction peaks around 30.5° (311), 36.3° (400, 43.4° (422), 56.9° (511), and 62.8° (440) corresponded to the crystalline structure of Fe_3_O_4_ (JCPDS file 19-0629) (Deng et al., 2009, 2019; Wang et al., 2017, 2018a,b; Munonde et al., 2018) These observations were in agreement with EDS and TEM results and other reports in the literature (Wang et al., 2017, 2018a,b).

**Figure 5:**
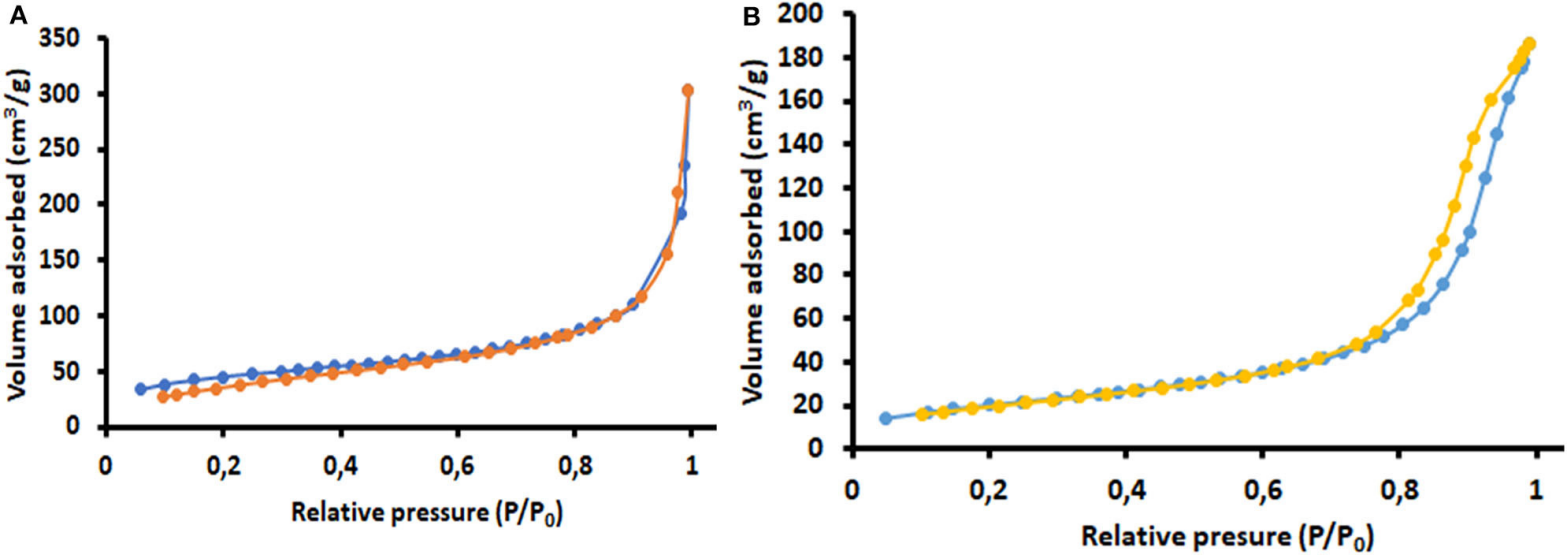
The Nitrogen adsorption-desorption isotherms for **(A)** P-POP and **(B)** MP-POP.

The surface properties of P-POP and MP-POP were investigated by N_2_ adsorption/desorption. The Brunauer-Emmett-Teller (BET) surface areas of the P-POP and MP-POP were found to be 478 and 295 m^2^ g^−1^, respectively. Moreover, the total pore volumes were 0.55 and 0.39 m^3^ g^−1^ for P-POP and MP-POP. It can be seen that the incorporation of Fe_3_O_4_ reduced the BET surface area of nanocomposite. According to Figure 5A, P-POP gives a typical type I isotherm signifying the classical characteristic of microporous materials (Cao et al., 2018; Wang et al., 2018b; Hao et al., 2019; Li et al., 2020). As shown in Figure 5B, the N_2_ adsorption-desorption isotherm for MP-POP present a distinctive type IV isotherm. These results reveal the presence of various pore sizes varying from micropores to mesopores (Wood et al., 2008; Nqombolo et al., 2019). In addition, large hysteresis loop at high relative pressure approaching 1.0, suggested the presence of microporous structures (Cao et al., 2018; Wang et al., 2018b; Hao et al., 2019; Nqombolo et al., 2019; Li et al., 2020).

### Selection of Eluent Type

**Figure 6:**
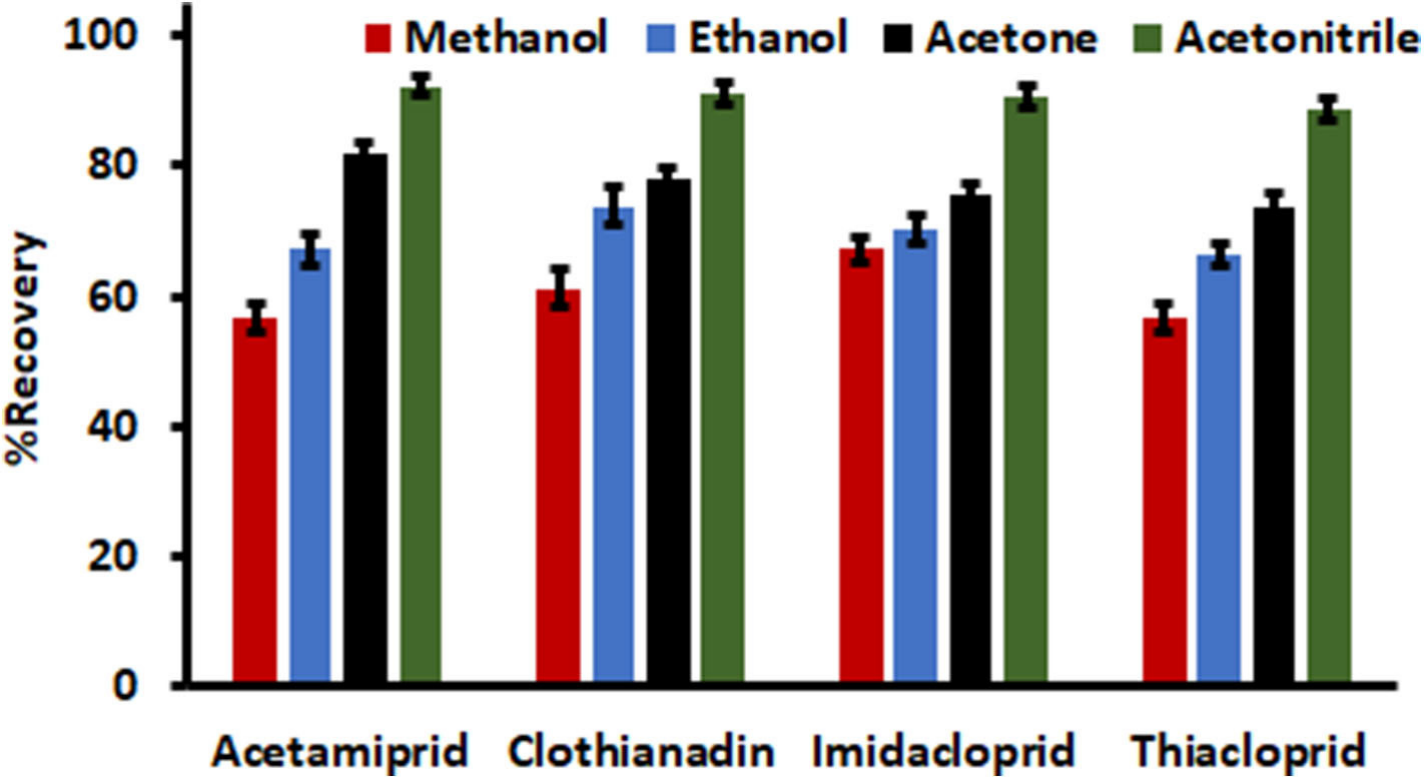
Selection of eluent type. Experimental conditions: sample volume, 5 mL; mass of adsorbent, 15 mg; acetonitrile volume, 750 μL; desorption time, 5 min; extraction time, 15 min and sample pH, 7.0.

The choice of a suitable elution solvent was investigated in order to achieved quantitative desorption of the analytes that are adsorbed on the surface adsorbent as well as attaining relatively high enrichment factor. In this study, the desorption capabilities of various HPLC grade organic solvents (ethanol, acetonitrile, acetone and methanol) were investigated and the adsorption-desorption experiments were carried in triplicate. The results in Figure 6 showed that the aprotic solvents (acetonitrile and acetone) had better elution capabilities as compared to methanol and ethanol. Hence, acetonitrile was chosen as the desorption solvent because it was the component of the mobile phase.

### Optimization Strategy

#### Fractional Factorial Design

A fractional factorial design (FFD) with four independent variables including pH of the sample, eluent volume (EV), mass of the adsorbent (MA) and extraction time (ET) at three levels which includes central points was used for the screening process. The design matrix and respective analytical response (percentage recovery) for each analyte are displayed (Supplementary Table 2). The data was assessed using analysis of variance (ANOVA). Pareto charts reproduced from the ANOVA results is used to indicate parameters that are significant for the preconcentration and extraction method. The blue bar on the left-hand side they represent the individual parameters and if the bar crosses the 95% confidence interval line (red line), it means that the parameter is significant (Zhang et al., 2017; Bezerra et al., 2019; Tan and Lee, 2019). As seen in Supplementary Figures 1A–D, the Pareto charts indicate that none of the parameters were insignificant at 95% confidence interval. Though, the bar length of EV, MA, and ET suggested that these three parameters has a significant role in the extraction and preconcentration of neonicotinoid insecticides. Therefore, EV, MA, and ET were further optimized using response surface methodology based on central composite design. The sample pH of the sample was fixed at 7.0.

#### Response Surface Methodology Based on Central Composite Design

Response surface methodology (RSM) based on CCD with three independent experimental parameters investigated at five levels, was used to investigate the interactions and quadratic effects of the main effects (Supplementary Table 3). The 3D surface plots demonstrating the effect of independent factors and their interactions on the analytical response are shown in Supplementary Figure 2. The influence of eluent volume (Supplementary Figures 2A,C) reveals that the quantitative recoveries were obtained between 600 and 1,200 μL of acetonitrile. Mass of adsorbent in solid phase extraction method plays a major role in achieving quantitative recoveries. As seen in Supplementary Figures 2A,B, mass of MP-POP as low as 15 mg could lead to extraction efficiency. Supplementary Figures 2B,C revealed that the effect of extraction lead to high extraction efficiency after 15 min.

#### Profile for Predicted Values and Desirability

The desirability function shown in Supplementary Figure 3, allows the simultaneous estimation of optimal values for all the investigated factors. Desirability always take values within 0-1 range where 0 indicates the least desired results (0.0%), 0.5 being the central point (50.8%), and 1 being the most desirable value assigned a percentage of (101.5%) (Candioti et al., 2014; Bezerra et al., 2019; Mashile et al., 2020). According to the desirability profiles, the optimal conditions for preconcentration and extraction process were MA of 15 mg, EV of 1,130 μL and extraction time of 15 min. The overall optimum parameters for extraction and preconcentration were 15 mg, 1,130 μL, 15 min and 7.0 for mass of the adsorbent, acetonitrile volume, extraction time and sample pH, respectively. The predicated optimum experimental parameters were verified experimentally. The results obtained for simultaneous extraction and preconcentration of four neonicotinoid insecticides ranged from 97.8 to 99.0%. The experimental findings were in full agreement with predicted values, suggesting that the model was valid. The obtained optimum conditions were used for further investigation.

### Effect of Initial Concentration and Sample Volume on the Recovery of Neonicotinoid Insecticides

Under optimized conditions, the influence of initial concentration on the percentage recovery of the selected neonicotinoid insecticides was assessed by processing various neonicotinoid insecticides solution at concentrations between 50 and 2000 μg L^−1^. The results in Supplementary Figure 4A, revealed that optimum recoveries (≥99%) at initial concentrations ranging from 50 to 1,200 μg L^−1^. Furthermore, the effect of sample volume was investigated in order to assess the possibility of achieving high preconcentration factors and examine its effect on percentage recoveries of the analytes. This was achieved by applying the optimized method to a series of sample solution ranging from 5 to 50 mL containing a fixed concentration of the analytes (100 μg L^−1^) of target analytes. The results obtained are presented in Supplementary Figure 4B and it can be seen that quantitative recoveries (>95%) of all four analytes was obtained for sample volumes of ≥ 30 mL. Therefore, the extracted analytes were could be eluted with 1,130 μL acetonitrile. The preconcentration factor of 27 was realized by the current method.

### Adsorption Capacity

Adsorption isotherms models are vital in describing and explaining interactive behavior of adsorbate and the adsorbent (MP-POP) (Bordoloi et al., 2017; Rafati et al., 2018). Therefore, equilibrium studies were carried in order to establish the adsorption mechanism. Equilibrium is established when the sample containing adsorbate is in contact with the absorbent for a specific time (Pezoti et al., 2016). The equilibrium data for the adsorption of neonicotinoid insecticides was done using various isotherms such as Langmuir, Freundlich, Redlich-Peterson and Dubin-Radushkevich models.

From the Table 1 below, isotherm models and parameters, the adsorption data followed Langmuir isotherm model with correlation efficiency of 0.9499, which was higher than that of Freundlich model for all the insecticides, this describes monolayer adsorption (Pezoti et al., 2016). Redlich-Peterson is a mixture of both Freundlich and Langmuir isotherms models, the focus here is at beta value, which is the exponent from the linear plot and is between 0 and 1 (Esfandiyari et al., 2017). When beta is equivalent to 1, the model reduces to Langmuir equation and when beta is 0 it reduces to Freundlich equation (Khare et al., 2018), from this study, the θ values were found to be 0.88 for acetamiprid, 0.65 for clothiandin, 0.75 for thiacloprid and 0.52 for imidacloprid which was ~1 suggesting the model reduces to Langmuir. The favoring of Langmuir by the isotherm models indicates that the adsorption was a monolayer adsorption. Dubin-Radushkevish model is normally used to differentiate between chemical and physical adsorption by looking at the mean energy (E) (Bordoloi et al., 2017; Rafati et al., 2018). The focus is on the energy value (E), if the value of energy is <8 kJ mol^−1^, then the adsorption was a physical one. If the energy value is among 8 and 16 kJ mol^−1^ then the adsorption was a chemical one (Bordoloi et al., 2017). In this study the calculated E values were 0.34, 0.22, 0.44, and 0.31 kJ mol^−1^ and for acetamiprid, cloathiandin, thiacloprid, and imidacloprid, respectively. The obtained energy values indicated physisorption processes.

**Table 1:** Isotherms equations and model parameters.

**Isotherms**	**Parameters**	**Acetamiprid**	**Clothiandin**	**Thiacloprid**	**Imidamiprid**
Langmuir $\frac{1}{q_{\;}}=\frac{1}{q_{\max}K_{L}}C_{e}+\frac{1}{q_{\max}}$	q_max_ (mg g^−1^)	99.0	85.5	90.0	79.4
	K_L_ (L mg^−1^)	0.10	0.11	0.09	0.16
	R^2^	0.9499	0.9286	0.9799	0.8767
Freundlich ln *q*_*e*_ = ln *K*_*F*_+ln *C*_*e*_	K	69.9	73.6	80.8	55.5
	N	3.0	2.0	2.0	2.5
	R^2^	0.9124	0.8926	0.8865	0.5621
Redlich-Peterson $\ln\;\left(K_{R}\cdot \frac{C_{e}}{q_{e}}-1\right)=b_{R}\ln\;C_{e}+\ln\;\alpha_{\text{R}}$	A	18.5	10.1	16.5	11.4
	B	0.88	0.65	0.75	0.52
	R^2^	0.8498	0.7140	0.6540	0.6281
Dubinin- Radushkevish $lnq_{e}=lnq_{m}-\beta E^{2}$	q_D−R_ (mg g^−1^)	67.7	57.1	51.6	60.7
	E (kJ mol^−1^)	0.34	0.22	0.44	0.31
	R^2^	0.8498	0.812	0.7219	0.7899

### Analytical Figures of Merit

The analytical performances of the established method for preconcentration and simultaneous extraction of neonicotinoid insecticides were investigated (Table 2), under optimum experimental conditions. The findings exhibited relatively wide linear dynamic ranges with coefficient of coefficients (*R*^2^) ranging from 0.9981 to 0.9994. The instrumental LODs and LOQs ranged from 1.3 to 3.2 ng L^−1^ and 4.3 to 11 ng L^−1^, respectively. The repeatability (intraday) and reproducibility (interday) expressed in terms of %RSD were <5% (Table 2), suggesting that the established method had relatively good precision. The preconcentration factor was estimated to be 27.

**Table 2:** Analytical characteristics of UA-DMSPE/HPLC-DAD method.

**Parameters**	**Clothianidin**	**Imidacloprid**	**Acetamiprid**	**Thiacloprid**
Linearity (μg L^−1^)	LOQ-550	LOQ-600	LOQ-450	LOQ-300
*R*^2^	0.9990	0.9983	0.9981	0.9994
LOD (ng L^−1^)	2.0	3.2	2.1	1.3
LOQ (ng L^−1^)	6.7	11	7.0	4.3
Intraday (%RSD), *n* = 10	1.3	1.5	1.2	1.8
Interday (%RSD), *n* = 5	2.7	3.5	2.6	4.1
Enrichment factor (EF)	104	94	98	110

*Experimental conditions: sample volume, 30 mL; mass of adsorbent, 15 mg; acetonitrile volume, 1,130 μL; time of desorption, 5 min; extraction time, 15 min and pH of the sample, 7.0*.

The development was also validated methods using spiked water samples. The linearity, LODs and LOQs and RSD for the studied analytes are presented in Table 3. The linearity ranging from LOQ-650 was obtained for the neonicotinoid insecticides. As seen the LODs and LOQs were ranged from 1.8 to 3.7 ng·L^−1^ and 6.0 to 12 ng·L^−1^, respectively. Whereas, the interday precision with spiked blank river water samples ranged from 2.1 to 6.1%. To evaluate and understand the effect of sample matrix on the extraction performance of the developed method, relative recoveries were tested by spiking ultra-pure water and river water samples. As seen, the relative recoveries of the target analytes were between 95 and 99%, suggesting that the current method did not suffer from matrix.

**Table 3:** Linearity, LODs and LOQs for target analytes in river water samples.

**Parameters**	**Clothianidin**	**Imidacloprid**	**Acetamiprid**	**Thiacloprid**
Linearity (μg L^−1^)	LOQ-600	LOQ-650	LOQ-550	LOQ-450
*R*^2^	0.9981	0.9912	0.9922	0.9953
LOD (ng L^−1^)	2.5	3.7	2.9	1.8
LOQ (ng L^−1^)	8.3	12	9.7	6.0
Intraday (%RSD), *n* = 10	2.1-5.3	3.4–5.9	3.5-5.6	3.3-6.1
Relative recoveries^a^	97.5 ± 3.7	98.3 ± 4.7	95.3 ± 4.6	98.3 ± 4.7

a Relative recoveries = (Concentration extracted in river/Concentration of each analyte extracted from ultrapure water) × 100.

*Experimental conditions: river sample volume, 30 mL; mass of adsorbent, 15 mg; acetonitrile volume, 1,130 μL; time of desorption, 5 min; extraction time, 15 min and pH of the sample, 7.0*.

The accuracy of the established method was investigated by analyzing spiked river water sample at two concentration points (Table 4) The river water samples were spiked with target analytes at three points (50, 100, and 500 ng L^−1^) and the samples were analyzed using the established method. The samples were also used to investigate the intraday and interday precision. As seen in Table 4, the percentage recoveries for the four target analytes ranged from 91 to 99.3% and the %RSD values ranged between 1.4 and 4.7%. The typical chromatogram of river spiked with 100 ng L^−1^ target is presented in Supplementary Figure 5. As seen the chromatogram shows good separation and there are no interfering peaks. This proves that the developed method was able to clean-up sample matrix, extract and preconcentrate the target analytes.

**Table 4:** Analysis of neonicotinoid insecticides in spiked river sample using UA-DMSPE/HPLC-DAD method.

**Insecticides**	**Added (ng L^**−1**^)**	**Measured (ng L^**−1**^)**	**Recovered (%R)**	**Intraday %RSD**	**Interday %RSD**
Clothiandin	0	8.10 ± 0.23	-	2.8	4.7
	50	56.9 ± 2.0	97.5	3.5	4.1
	100	107 ± 5	98.5	1.9	3.2
	500	503 ± 7	99.0	1.4	3.7
Imidacloprid	0	42.3 ± 0.7	-	1.8	3.2
	50	91.7 ± 2.4	98.8	2.6	3.5
	100	142 ± 0.08	99.3	2.1	2.5
	500	538 ± 9	99.1	1.7	2.7
Acetamiprid	0	4.85 ± 0.12	-	2.5	4.5
	50	51.8 ± 1.3	93.8	2.5	3.4
	100	99.4 ± 2.3	94.5	2.3	3.1
	500	483 ± 9	95.6	1.9	2.2
Thiacloprid	0	< LOD	-	-	-
	50	45.3 ± 1.3	90.5	2.9	4.1
	100	94.6 ± 3	94.6	3.2	3.7
	500	477 ± 10	95.4	2.1	3.5

*Experimental conditions: sample volume, 30 mL; mass of the adsorbent, 15 mg; acetonitrile volume, 1,130 μL; time of desorption, 5 min; extraction time, 15 min and pH of sample, 7.0*.

### Comparison of the Developed Method With Others Reported in the Literature

The analytical figures of merit for the developed method were compared with those that were previously reported for preconcentration and extraction of clothianidin, imidacloprid, acetamiprid, and thiacloprid their determination using chromatographic techniques are displayed in Table 5. As seen, the established method had lower LODs and LOQs compared those reported by elsewhere (Sánchez-Hernández et al., 2014; Bolzan et al., 2015; Cao et al., 2018; Kachangoon et al., 2020). The relative standard deviation was found to be better than those reported in literature (Table 5). In addition, the LODs and LODs were comparable (in the same magnitude) with those reported in the literature (Zhang et al., 2017; Moyakao et al., 2018; Xiong et al., 2018). However, the LODs and LOQs were greater than those reported elsewhere (Li and Shi, 2019). Lastly, the method proved to have attractive advantages such as good sensitivity and simplicity because of the low LOD and LOQ, high precision and wide linearity. Even though, the performance of the MPOP was comparable to traditional SPE adsorbent such as HLB/GCB (Zhang et al., 2017) as well as HLB combined with C18 (Dujaković et al., 2010), these method combined two types of traditional adsorbent in order to achieve low LODs and LOQs. As in Table 5, studies have proven that when C18 is used alone, the high LODs were between 1.0 and 2.3 μg L^−1^ were obtained (Sánchez-Bayo and Hyne, 2014). Therefore, the advantages of the developed method over the traditional SPE procedures include short preconcentration time (15 min extraction and 5 min desorption time), use of a single easily recoverable adsorbent with high surface area, easy operation and low cost. Moreover, the proposed method is environmentally friendly compared to traditional SPE because it uses small amount of a reusable adsorbent (15 mg) and the use of excessive organic solvents in minimized (in this work only 1,130 μL was used).

**Table 5:** Comparison of analytical performance of the developed method with others that are reported in literature.

**Analytes**	**Sample matrix**	**Method**	**Adsorbent**	**LDR (μg L^**−1**^)**	**LOD (μg L^**−1**^)**	**LOQ (μg L^**−1**^)**	**%RSD**	**References**
Clothiandin, imidacloprid, acetamiprid, thiacloprid	Water	DSPE-HPLC–MS	MOF(UIO-66)	10–500	0.02–0.4	0.05–1.0	8.5–13.1	Cao et al., 2018
Clothiandin, imidacloprid, acetamiprid, thiacloprid	Beewax	CE–ESI-MS	C18	LOQ-1,000	1.0–2.3	3.3–7.7	1.37–3.5	Sánchez-Hernández et al., 2014
Clothiandin, imidacloprid, acetamiprid, thiacloprid	Water	HPLC/MS/MS	HLB/GCB		0.0018–0.0045	0.006–0.015	5.3–12	Zhang et al., 2017
Clothianidin, imidacloprid, thiacloprid	Water	CPE/HPLC-UV	—	1–1000	0.3–1.0	1.0–3.3	<10	Kachangoon et al., 2020
Clothiandin, imidacloprid, acetamiprid, thiacloprid	Water	SPE/HPLC–MS/MS	CNT	0.00025–0.1	0.0001	0.00025–0.00005	2.4–12.2	Li et al., 2019
Acetamiprid, imidacloprid	water	SPE-LC-MS/MS	HLB & C18		0.0004–0.0055	0.0013–0.0017	4–23	Dujaković et al., 2010
Clothiandin, imidacloprid, acetamiprid, thiacloprid	Water	SPE- HPLC–MS/MS	Montmorillonite		0.0018–0.013	0.006–0.043	<20	Xiong et al., 2018
Thiamethoxam, imidacloprid, acetamiprid	Surface water	VA-d-μ-SPE/HPLC-PDA	–	0.5–1,000	0.005–0.065	0.008–0.263	2.8–7.1	Moyakao et al., 2018
Imidacloprid	Mineral water	DLLME		0.5–1.5	0.15	0.5	1–3%	Bolzan et al., 2015
Clothiandin, imidacloprid, acetamiprid, thiacloprid	River water	UA-DMSPE-HPLC-DAD	MP-POP	LOQ-600	0.0013–0.0032	0.0043–0.011	1.4–4.7	This work.

*DSPE, Dispersive solid phase extraction; HPLC, high performance liquid chromatography; CE, ESI, CPE MS= mass spectroscopy, VA-d- μ, vortex assisted-dispersive micro solid phase extraction; DLLME, dispersive liquid-liquid microextraction; LC, liquid chromatography; PDA, photodiode array; UV, ultraviolet; DAD, diode array detector*.

### Application to Real Water Samples

The applicability of MP-POP as a sorbent for extraction and preconcentration of neonicotinoid insecticides was carried out by the analysis of two river samples. As seen, trace amounts of target analytes were detected in river water samples (Table 6, Supplementary Figure 6) except thiacloprid in river sample 1. Clothiandin, acetamiprid and imidacloprid were frequently detected in all river samples at relatively higher concentrations (7.5–8.10 ng L^−1^ clothiandin, 4.6–109 ng L^−1^ imidacloprid and 4.85–20.7 ng L^−1^ acetamiprid) compared to thiacloprid. This is because these three neonicotinoid insecticides are the mostly used in tomato and maize plantation which are common in South Africa. The results showed that MP-POP can be applicable for extraction and preconcentration of neonicotinoid insecticides in real samples regardless of the complex matrix. This is shown by the smoothness of chromatograms that there was no interference from the complex matrix of the samples (Supplementary Figure 6).

**Table 6:** Concentration of neonicotinoid insecticides in river water samples obtained using UA-DMSPE/HPLC-DAD method.

**Samples**	**Clothiandin**	**Imidacloprid**	**Acetamiprid**	**Thiacloprid**
River 1	7.49 ± 0.11	109 ± 4	20.7 ± 0.9	ND
River 2	8.02 ± 0.27	4.64 ± 0.22	14.0 ± 0.7	8.43 ± 0.31

*Experimental conditions: sample volume, 30 mL; mass of adsorbent, 15 mg; acetonitrile volume, 1,130 μL; time of desorption, 5 min; extraction time, 15 min and pH of the sample, 7.0*.

The maximum concentration of neonicotinoid insecticides obtained in this study were compared with those reported in the literature globally (Table 7). As see the concentration levels of neonicotinoid insecticides in this study were lower than those reported in United States (Starner and Goh, 2012; Ensminger et al., 2013), Canada (Main et al., 2014; Schaafsma et al., 2015), Benin (Berny's et al., 2019), and China (Zhang et al., 2017, 2019). They were higher than those reported in Japan (Yamamoto et al., 2012) and China (Xiong et al., 2018).

**Table 7:** Global concentrations of neonicotinoid insecticides in river water samples.

**Country**	**Concentration range (ng L^**−1**^)**	**References**
China	4.47–52.4	Xiong et al., 2018
US	0–3,290	Starner and Goh, 2012
Canada	0–173	Main et al., 2014
China	0–193	Zhang et al., 2017
US	50-160	Ensminger et al., 2013
China	6.24–154	Zhang et al., 2019
Canada	40–5,950	Schaafsma et al., 2015
Japan	0–25	Yamamoto et al., 2012
Benin	200–7,700	Berny's et al., 2019
South Africa	0–109	This study

### Reusability and Regeneration

The adsorbent regeneration and reusability were investigated by a series of extraction, elution, washing and drying. After each extraction the percentage recovery was calculated, and the findings are illustrated in Supplementary Figure 7. The percentage recoveries showed a significant decrease after cycle number 6. The results found demonstrated that recovery and adsorption for neonicotinoid insecticides were not affected for up to five cycles. Therefore, this showed a great reusability of the synthesized adsorbent and also showed its excellent regeneration properties. The significant decrease could be due to the collapsing of the pores of MP-POP and as the adsorbent was continuously used, it lost its affinity toward the neonicotinoid insecticides. The loss of affinity was also caused by the multiple washing of the adsorbent. Multiple washing of the adsorbent leads to the deterioration of the functional groups which are responsible for analyte binding on the adsorbent.

### Adsorption Mechanism of the M-PPOP

The conceivable mechanism for the adsorption of neonicotinoid insecticides on the surface of the adsorbent was investigated following the method reported in the literature (Wang et al., 2017, 2018a). According to the literature, EF of each analyte can be used to investigate the adsorption affinity of the MP-POP toward the analytes on interest (Wang et al., 2017, 2018a,b). As seen, in the Supplementary Table 6, relatively high EFs (94–110) were obtained for the four investigated neonicotinoid insecticides. In addition, hydrophobicity indicator (Log K_ow_), hydrogen bonding preference indicators (that is, H bond acceptors and donors) were used to evaluated the adsorption mechanism (Wang et al., 2017, 2018a). As seen in Supplementary Table 4, the Log K_ow_ (Chevillot et al., 2017; Sultana et al., 2018) was as follows: imidacloprid > clothianidin> acetamiprid > thiacloprid. This order suggested that for analytes with relatively high Log K_ow_ like thiacloprid and acetamiprid the hydrophobic and π-staking interactions between the adsorbate and the adsorbent played a vital part during the extraction and preconcentration process (Wang et al., 2017, 2018a). Hydrogen bonding interaction had a major influence in the adsorption of imidacloprid and clothianidin. This because these two analytes have higher number hydrogen-bonding donor and acceptor sites as compared to imidacloprid and clothianidin (Supplementary Table 6). In view of the above, it was concluded that the extraction and preconcentration of neonicotinoid insecticides using MP-POP nanocomposite was driven by hydrogen bonding, hydrophobic and π-stacking interactions. These findings are alike to those reported in the literature (Wang et al., 2017, 2018a,b).

## Conclusions

A rapid, simple, reliable and efficient UA-DMSPE/HPL-DAD method was developed for the simultaneous extraction, preconcentration determination of acetamiprid, clothiandin, thiacloprid, and imidacloprid in river water samples. The MP-POP adsorbent displayed relatively high chemical and thermal stabilities, remarkable regeneration and reusability properties as well as hig affinity torward target analytes. Coupling of HPLC-DAD with the preconcentration method resulted in improved LODs, LOQs, linear dynamic range, sensitivity, precision, accuracy and acceptable recoveries. As the result, the analytical performance of UA-DMSPE/HPL-DAD method was comparable to those found using LC-MS/MS. The applicability of the UA-DMSPE/HPL-DAD method was evaluated by analyzing the target analytes in spiked river water samples and percentage recovery values ranged from 91 to 99%. Furthermore, the method was later applied for determination of acetamiprid, clothiandin, thiacloprid, and imidacloprid in river water samples and the concentrations were lower or comparable with those detected in other countries. These results proved that the established method could be used for determination of neonicotinoid insecticides in complex matrices.

## Data Availability Statement

The raw data supporting the conclusions of this article will be made available by the authors, without undue reservation.

## Author Contributions

SS and PN: conceptualization. SS: methodology, investigation, methodology, validation, and writing- original draft preparation. AM, SS, and NW: data curation, sampling, reviewing, and editing. PN: software, funding acquisition, supervision, validation, writing- reviewing, and editing.

## Conflict of Interest

The authors declare that the research was conducted in the absence of any commercial or financial relationships that could be construed as a potential conflict of interest.
