# Supplementary material for: Magnetic Solid Phase Extraction Based on Nanostructured Magnetic Porous Porphyrin Organic Polymer for Simultaneous Extraction and Preconcentration of Neonicotinoid Insecticides From Surface Water
Source: Front Chem. 2020 Sep 16;8:555847. doi: 10.3389/fchem.2020.555847 (PMC7525214; doi:10.3389/fchem.2020.555847)
Supplement: Supplementary file 1 [file Table_1.DOC]

**Magnetic solid phase extraction based on nanostructured magnetic porous porphyrin organic polymer for simultaneous extraction and preconcentration of neonicotinoid insecticides from surface water**

**Shirley K. Selahle^1,2^, Ngwako J. Waleng^1,2^, Anele Mpupa^1.2^, Philiswa N. Nomngongo1,2,3[[1]](#footnote-2)**

*^1^ Department of Chemical Sciences, University of Johannesburg, Doornfontein Campus, P.O. Box 17011, Doornfontein, 2028, South Africa*

*^2^DSI/NRF SARChI Chair: Nanotechnology for Water, University of Johannesburg, Doornfontein 2028, South Africa.*

*^3^DSI/Mintek Nanotechnology Innovation Centre, University of Johannesburg, Doornfontein 2028, South Africa*

Supplementary Material

# Supplementary Figures and Tables

## Supplementary Tables

Table S1: Independent variable and their levels used in design of experiments (DOE)

| **Variable** | **Minimum** | **Central** | **Maximum** |
| --- | --- | --- | --- |
| **Mass of adsorbent (mg)** | 20 | 25 | 50 |
| **pH** | 4 | 6.5 | 9 |
| **Extraction time (min)** | 10 | 15 | 20 |
| **Eluent volume (µL)** | 100 | 550 | 1000 |

Table S2: Factorial design matrix and the respective analytical responses

| **Standard Run** |  | | | | |  |  |  |
| --- | --- | --- | --- | --- | --- | --- | --- | --- |
|  | MA | EV | pH | ET | Clothi | Imida | Aceta | Thia |
| **1** | 20 | 100 | 4 | 10 | 43,6 | 23,9 | 28,4 | 31,3 |
| **2** | 50 | 100 | 4 | 20 | 18,4 | 16,4 | 17,3 | 20,7 |
| **3** | 20 | 1000 | 4 | 20 | 88,9 | 65,4 | 74,7 | 78,1 |
| **4** | 50 | 1000 | 4 | 10 | 67,2 | 36,6 | 43,1 | 42,9 |
| **5** | 20 | 100 | 9 | 20 | 37,8 | 47,9 | 55,1 | 46,3 |
| **6** | 50 | 100 | 9 | 10 | 48,6 | 20,5 | 27,6 | 23,3 |
| **7** | 20 | 1000 | 9 | 10 | 78,8 | 45,2 | 51,0 | 53,2 |
| **8** | 50 | 1000 | 9 | 20 | 78,0 | 40,2 | 44,2 | 52,9 |
| **9 (C)** | 35 | 550 | 7 | 15 | 82,0 | 72,0 | 81,7 | 83,7 |
| **10 (C)** | 35 | 550 | 7 | 15 | 84,6 | 68,4 | 84,2 | 85,5 |
| **11 (C)** | 35 | 550 | 7 | 15 | 82,1 | 72,4 | 83,8 | 85,3 |
| **12 (C)** | 35 | 550 | 7 | 15 | 82,2 | 69,1 | 87,0 | 87,8 |

Table S3: Central composite design matrix and the respective analytical responses.

| **Expt** | **Mass of adsorbent** | **Eluent volume** | **Extr. time** | **%R** | | | |
| --- | --- | --- | --- | --- | --- | --- | --- |
|  |  |  |  | Aceta | Chlothia | Imida | Thia |
| **1** | 20.0 | 100 | 10 | 55.5 | 26.0 | 32.7 | 35.7 |
| **2** | 20.0 | 100 | 20 | 76.6 | 69.7 | 80.3 | 92.6 |
| **3** | 20.0 | 1000 | 10 | 103.0 | 95.0 | 102.1 | 97.8 |
| **4** | 20.0 | 1000 | 20 | 87.8 | 100.3 | 89.3 | 97.3 |
| **5** | 50.0 | 100 | 10 | 66.1 | 55.1 | 63.5 | 86.1 |
| **6** | 50.0 | 100 | 20 | 98.2 | 92.5 | 106.3 | 95.8 |
| **7** | 50.0 | 1000 | 10 | 95.5 | 100.7 | 79.9 | 70.6 |
| **8** | 50.0 | 1000 | 20 | 78.3 | 84.4 | 97.6 | 80.2 |
| **9** | 15.7 | 550 | 15 | 26.4 | 24.7 | 27.6 | 32.2 |
| **10** | 54.0 | 550 | 15 | 40.2 | 58.0 | 18.2 | 31.7 |
| **11** | 35.0 | -29 | 15 | 0.0 | 0.0 | 0.0 | 0.0 |
| **12** | 35.0 | 1129 | 15 | 83.4 | 82.1 | 82.1 | 99.0 |
| **13** | 35.0 | 550 | 8.6 | 69.5 | 65.5 | 65.5 | 73.2 |
| **14** | 35.0 | 550 | 21 | 70.0 | 81.8 | 81.8 | 81.5 |
| **15 (C** | 35.0 | 550 | 15 | 96.2 | 97.8 | 97.8 | 94.9 |
| **16 (C)** | 35.0 | 550 | 15 | 96.1 | 101.5 | 101.5 | 97.4 |

Table S4 Physicochemical properties of neonicotinoid insecticides and their EF values on the MP-POP adsorbent

| **Compound name** | **Structures** | **Molecular mass (g/mol)** | **LogK_ow_^a^** | **EF** | **H bond acceptor** | **H bond donors** |
| --- | --- | --- | --- | --- | --- | --- |
| **Acetamiprid** | 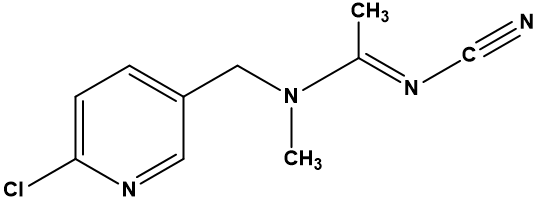 | 222.67 | 0.8 | 98 | 4 | 0 |
| **Imidacloprid** | 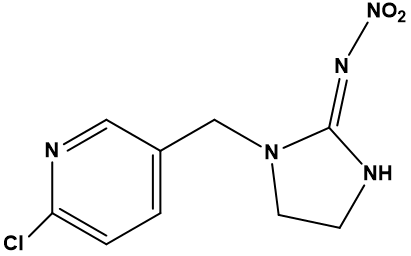 | 255.661 | 0.6 | 94 | 5 | 1 |
| **Clothianidin** | 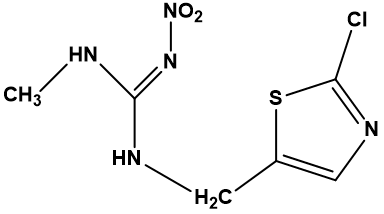 | 249.678 | 0.7 | 104 | 5 | 2 |
| **Thiacloprid** | 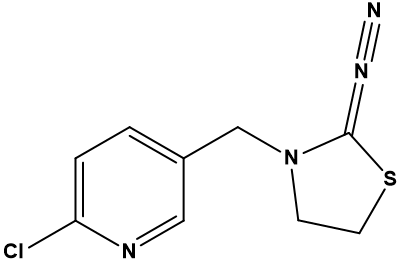 | 252.72 | 1.3 | 110 | 4 | 0 |

^a^ Data adopted from

## Supplementary Figures


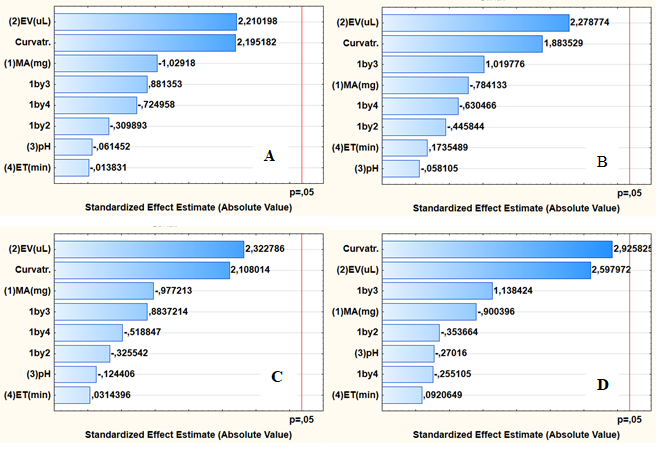


Figure S1. Pareto charts of standardised effect for preconcentration of (a) acetamiprid, (b) clothianadin, (c) imidacloprid and (d) thiacloprid. EV = Eluent volume; MA= Mass of adsorbent; ET=; Extraction time, 1 by 2= interaction of mass and the eluent volume; 1by3 = interaction of mass of adsorbent and pH; 1 by 4 = Interaction of mass of the adsorbent and extraction time;


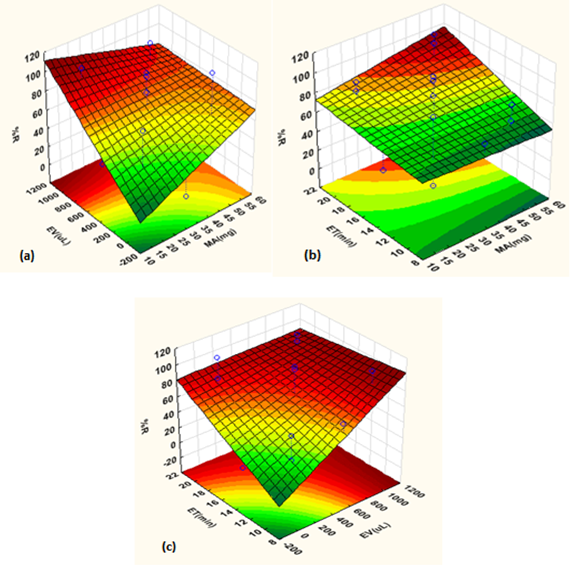


Figure S2. Response surface methodology 3D surface plots showing interaction effects.


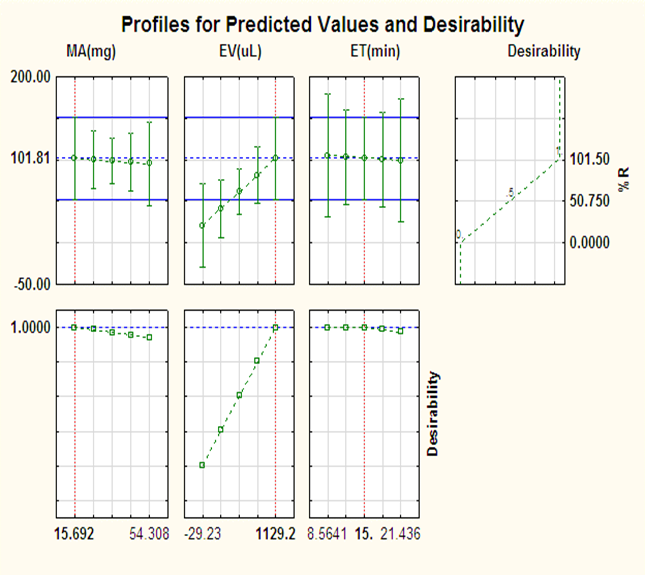


Figure S3. Profile for predicted values and desirability


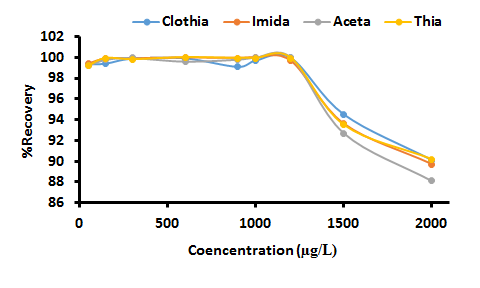

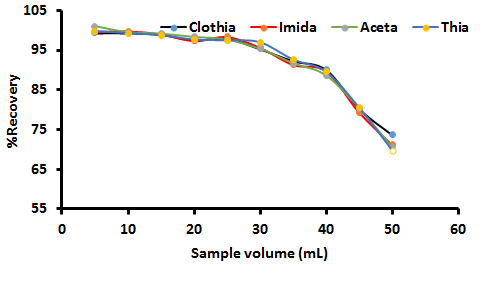


**A**

**B**

Figure S4 Effect initial concentration and sample volume


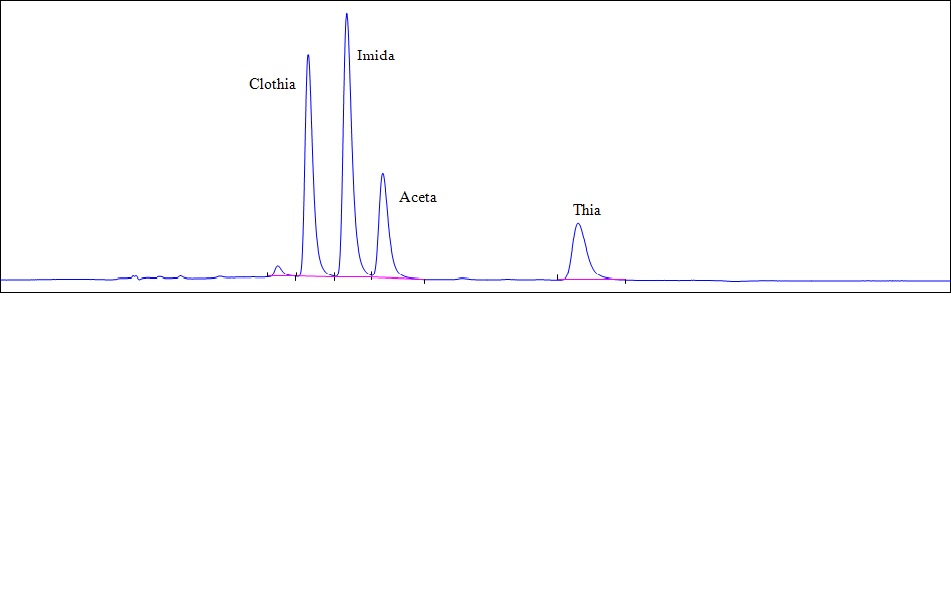


Figure S5. Chromatogram of acetamiprid (Aceta), imidacloprid (Imida), Clothiandin (Clothia) and Thiacloprid (Thia) from river water sample spiked with 100 ng L^−1^ of each analyte.


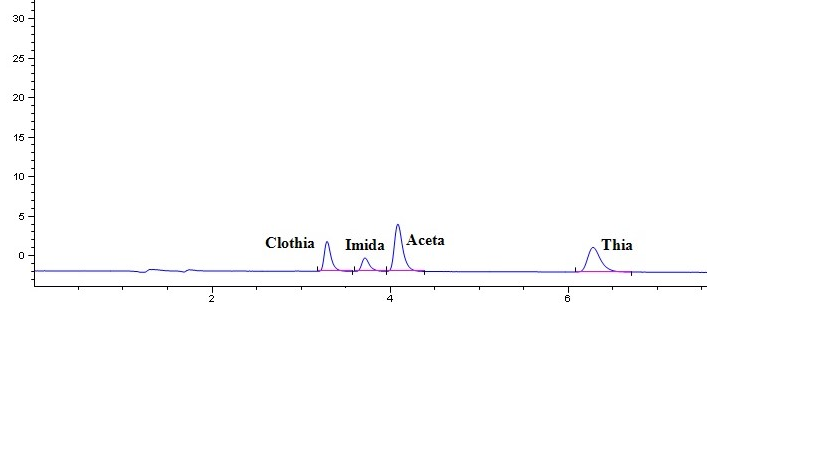


Figure S6. Typical chromatogram obtained after application of UA-DMSPE/HPLC-DAD method for analysis of river water sample 1. Acetamiprid (Aceta), imidacloprid (Imida), Clothiandin (Clothia) and Thiacloprid (Thia)


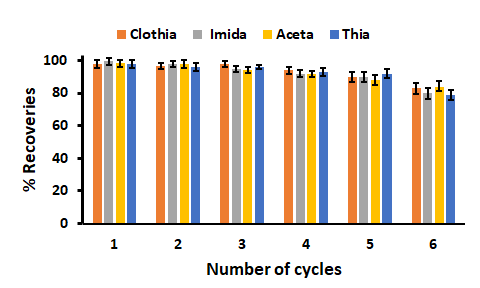


Figure S7. Regeneration and reusability of MP-POP. Acetamiprid (Aceta), imidacloprid (Imida), Clothiandin (Clothia) and Thiacloprid (Thia). Experimental conditions: sample volume, 30 mL; mass of the adsorbent, 15 mg; acetonitrile volume, 1130 µL; time of desorption, 5 min; extraction time, 15 min and pH of sample, 7.0.

1. Corresponding author: E-mail address:pnnomngongo@uj.ac.za, Tel: +27115596187 [↑](#footnote-ref-2)
